# Positivity rate, trend and associated risk factors of mother-to-child transmission of HIV among HIV-exposed infants

**DOI:** 10.1186/s12887-023-04074-2

**Published:** 2023-06-06

**Authors:** Gadissa Gutema, Habteyes Hailu Tola, Dinka Fikadu, Dereje Leta, Birra Bejiga, Jaleta Bulti Tura, Saro Abdella, Hassen Mamo

**Affiliations:** 1 HIV/AIDS Research Team, TB and HIV/AIDS Research Directorate, Ethiopian Public Health Institute, PO Box 1242, Addis Ababa, Ethiopia; 2 Department of Microbial, Cellular and Molecular Biology, College of Natural and Computational Sciences, Addis Ababa University, PO Box 1176, Addis Ababa, Ethiopia; 3 College of Health Sciences, Salale University, Fiche, Ethiopia; 4 TB Research Team, TB and HIV/AIDS Research Directorate, Ethiopian Public Health Institute, Addis Ababa, Ethiopia

**Keywords:** Human immunodeficient virus, Early Infant Diagnosis, MTCT, HIV-exposed infants

## Abstract

**Background:**

Mother-To-Child-Transmission (MTCT) of Human Immunodeficiency Virus (HIV) occurs during pregnancy, delivery and breastfeeding, and cause infection among several new-borns. However, there is limited recent evidence on the burden of MTCT of HIV in Ethiopia from a large-scale data. Thus, this study aimed to determine the positivity rate, trend and associated risk factors of MTCT among HIV-exposed infants.

**Methodology:**

A cross-sectional study was conducted among 5,679 infants whose specimen referred to Ethiopian Public Health Institute HIV referral laboratory for Early Infant Diagnosis (EID) from January 01, 2016, to December 31, 2020. Data were extracted from the national EID database. Frequencies and percentages were used to summarize the data on characteristics of infants. Logistic regression analysis was employed to identify factors associated with positivity rate of MTCT of HIV. Level of significance was set at 5%.

**Results:**

The mean age of the infants was 12.6 (± 14.6) weeks with an age range of 4 to 72 weeks. Half of the infants (51.4%) were female. The positivity rate of MTCT decreased from 2.9% in 2016 to 0.9% in 2020 with five-year average positivity rate of 2.6%. HIV test after six weeks (Adjusted odds ratio (AOR) = 2.7; 95% confidence interval (CI): (1.8–4.0,)); *p* < *0.001)*, absence of prevention of mother-to-child-transmission (PMTCT) service (AOR = 4.6; 95% CI: (2.9–7.4)); *p* = *0.001)*, nevirapine prophylaxis not received (AOR = 2.0; 95% CI: (1.3–3.2)); *p* < *0.001*), and unknown ART status of the mother at delivery (AOR = 11; 95% CI: (5.5–22.1)); *p* < *0.001)* were significantly associated with MTCT of HIV.

**Conclusion:**

The positivity rate of MTCT of HIV was showing declining tendency gradually in the study period. Strengthening PMTCT service, early HIV screening and starting ART for pregnant women, and early infant diagnosis are required to reduce the burden of HIV infection among infants exposed to HIV.

## Background

Human Immunodeficiency Virus (HIV) can be transmitted from HIV-positive mothers to their babies during pregnancy, at delivery and during breastfeeding which accounts for the majority of infection in children [1–3]. The proportion of Mother-To-Child-Transmission (MTCT) of HIV in treatment–naive pregnant women range from 15 to 45% [2, 4]. However, antiretroviral therapy (ART), post-exposure prophylaxes and Prevention of Mother-To-Child Transmission (PMTCT) services significantly reduced the proportion of MTCT to less than 5% [2, 4]. Virological screening for exposed infants at six weeks of age or the earliest possible stage after six weeks plays a great role in PMTCT [2, 5, 6]. However, only half of HIV-exposed infants (HEIs) are tested within the World Health Organization (WHO) recommended age [7].

Although different efforts being made to prevent MTCT of HIV, about 400 children under 15 years of age infected with HIV per a day in 2020 globally [8]. In the same year, it was estimated that 1.7 million children live with HIV, while 99,000 children died due to Acquired Immune Deficiency Syndrome (AIDS), and 150,000 acquired new HIV infection [8]. Moreover, each year over half a million new born are infected with HIV in sub–Saharan Africa countries through MTCT [9].

MTCT is generally declining across the world [10]. For instance, in Vietnam the prevalence of MTCT declined from 27.9% in 2007 to 0% in 2018 [11]. Moreover, the prevalence of HIV transmission decreased from 10.4% in 2006 to 0% in 2015 in Burkina Faso [12]. The prevalence of MTCT is also declined from 17.0% in 2007 to 7.2% in 2013 in Kenya [13]. It is also showing declining trend in Ethiopia [8]. An estimate indicates that, 6,200 children were newly infected with HIV in 2010, while 2,700 in 2018 [8] which shows significant reduction.

Despite the fact that ART for children was launched in 2005 in Ethiopia, the national PMTCT program began in 2001 [14]. The PMTCT program includes the provision of HIV care/ART for mothers, ARV prophylaxis for mothers and infants, and infant feeding counselling. Evidence shows, 92% of pregnant women living with HIV have access to antiretroviral treatments in 2020 in Ethiopia [15]. The MTCT prevalence declined by half in 2020 compared to 2010 (15% versus 32.3%) in Ethiopia [15], Laboratory test coverage for Early Infant Diagnosis (EID) was also declined from 48.5% in 2010 to 40.9% in 2020 [15]. Although the prevalence of MTCT of HIV is declining in Ethiopia, some studies indicate that considerable number of infants born from HIV-positive mothers are infected with the virus [14–17]. For instance, 10.1% in 2014 [16], 5.8% in 2019 [18], and 2.3% in 2020 [15] infants born from HIV-positive mothers were infected with HIV in Amhara region. Moreover, 2% of infants are infected by HIV in Addis Ababa [19]. A systematic review and meta-analysis studies pooled data reported from different parts of Ethiopia indicated 11.4% and 9.9% of infants HIV infected through MTCT [20, 21].

Several factors are associated with HIV infection among infants born from HIV positive mothers. For example, delayed diagnosis, failure to receive either ART or prophylaxis during pregnancy, breastfeeding, and short duration since prophylaxes started during pregnancy are factors significantly associated with HIV infection among infants born from positive mothers [17]. Moreover, infants not receiving ART prophylaxis at birth, mixed feeding practices, and mother–child pairs not receiving prophylaxes are also significantly associated with MTCT of HIV [19].

Global recommendation indicates elimination of new HIV infections among children in every country. However, Ethiopia is among the ten countries with the highest burden of HIV infections among children due to considerable MTCT of the virus [4]. Although there are small scale local studies that reported the prevalence of HIV among infants born from positive mothers in Ethiopia, there is limited evidence generated from large scale samples to inform policy-makers and program officers. Thus, this study was aimed to estimate the positivity rate, past five-year trend and factors associated with HIV infection among infants born from HIV positive mothers in Ethiopia.

## Methods

**Fig. 1:**
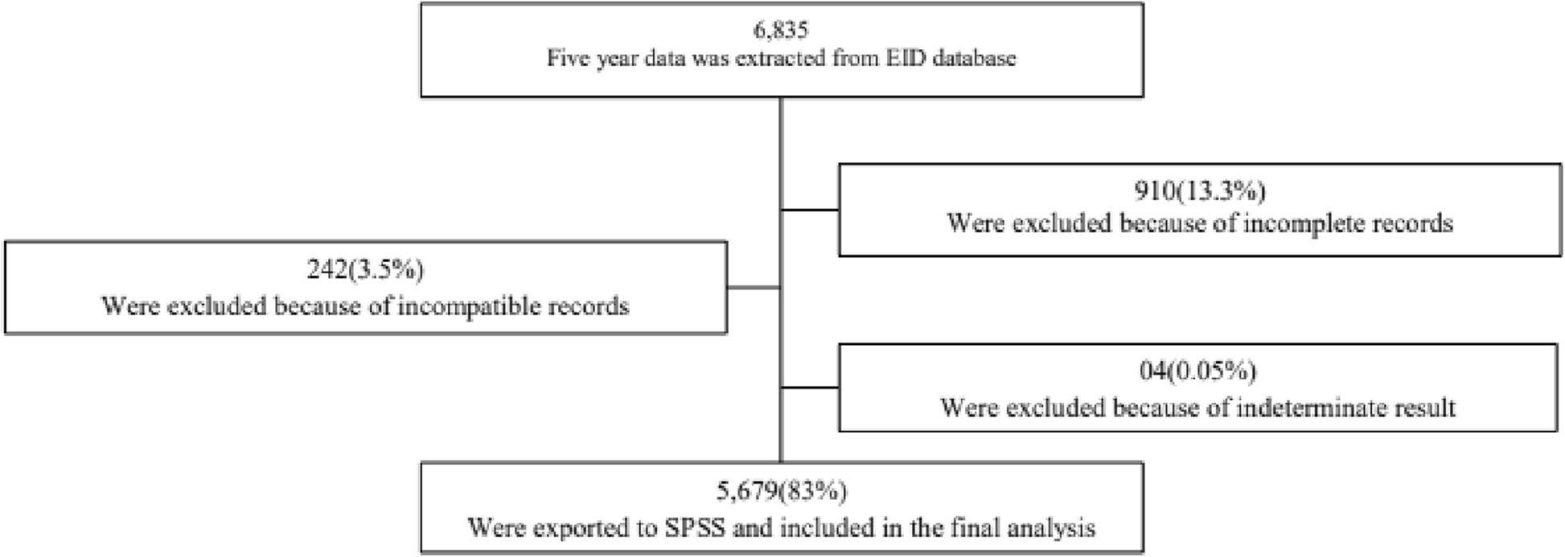
Data extraction work flow

A cross-sectional study was conducted to determine the positivity rate, trend, and factors associated with HIV infection among infants born from HIV-positive mothers in Ethiopia. This study was conducted in Ethiopian Public Health Institute (EPHI) national HIV referral laboratory. EPHI is also a national biomedical and public research institute and provide referral laboratory service for the nation. National HIV referral laboratory is one of the biomedical laboratories found in EPHI, and the molecular laboratory in HIV referral laboratory has obtained ISO 15189 accreditation certificate in 2012 by Ethiopian national accreditation office. The national accreditation office is the only mandated organization to accredit the laboratories in the country by the government of Ethiopia. As it is the national reference laboratory it receives specimens for HIV EID from every part of the country when the service is interrupted in other laboratories in the country due to reagent stock out and problems related to machine. Thus, national HIV reference laboratory conduct EID testing from Dried Blood Spot (DBS) specimens for the past 16 years. Data on all infants born from HIV-positive mothers and whose specimens referred to EPHI HIV national referral laboratory for EID from January 01, 2016 to December 31, 2020 were included to this study analysis (Fig. 1).

### Inclusion and exclusion criteria

Children with HIV test results and information on key variables were included in this study data analysis. However, children with incomplete record and indeterminate results were excluded.

### Sample size

A total of 6,835 specimens of infants born from HIV-positive mothers were sent to the EPHI HIV referral laboratory for EID tests in the past five years (from 2016–2020). Of these, a total of 5,679 infants with complete data were included in this study data analysis (Fig. 1).

### Data collection

The variables on which data was extracted were infant sex, age (in week), HIV status, feeding practice, mother ART status at delivery, and PMTCT services status provided for infants and mothers. The outcome variable was HIV infection which was confirmed by automated Polymerase Chain Reaction (PCR) using COBAS AmpliPrep/COBAS TaqMan (Roche CAP/CTM) HIV–1 proviral Deoxyribonucleic Acid (DNA) qualitative detection technique [22].

### Statistical analyses

The data was reviewed and checked for completeness. Descriptive statistics was used to show participants distribution. A logistic regression model was applied to determine factors associated with HIV infection among infants. Variables scored *p-*value < 0.2 during bivariate analysis were included in the multivariate analysis model by stepwise model building methods. Odds ratios (ORs) with 95% confidence intervals (CIs) were reported to show strength of association and level significance was set at 5%.

## Results

### General characteristics of study participants

A total of 5,679 HEIs data were included in this study analysis. Half (51.4%) of the infants were female and 57% born from Addis Ababa residents. The mean age of the infants was 12.6(± 14.6) weeks with an age range of 4—72 weeks. The proportion of infants tested for HIV at WHO recommended age (age ≤ 6 weeks) was 56.5% (Table 1). PMTCT service was provided for 95.4% of enrolled infants who born from HIV positive mothers. The majority (95.3%) of infants received nevirapine prophylaxis at delivery and for six weeks after birth.

**Table 1:** Socio–demographic and general characteristics of HIV exposed infants in Ethiopia, 2016 to 2020

Variable	Frequency	Percentage
Sex		
Female	2921	51.4
Male	2758	48.6
Age		
≤ 6 weeks	3206	56.5
> 6 weeks	2473	43.5
Infant feeding practice		
Exclusive breast feeding	4150	73.1
Replacement feeding	1529	26.9
Intervention given to infant in PMTCT		
Present	5420	95.4
Absent	259	4.6
Infant received nevirapine prophylaxis therapy		
Yes	5166	95.3
No	254	4.7
Mother ART status during delivery		
Newly initiated upon delivery	1478	26.0
Previously on ART	4145	73.0
Status unknown	56	1.0

Intervention given to infant includes Nevirapine prophylaxis, Cotrimoxazole prophylaxis, and counselling on infant -feeding practice

*PMTCT* Prevention of Mother-to-Child-Transmission, *ART* Antiretroviral Therapy

### HIV Positivity rate among HEI and regional distribution

The overall positivity rate of HIV infection over the past-five year among infants born from HIV positive mothers was 2.6%. The highest positivity rate (3.9%) of HIV infection was observed in the Oromia region and the lowest (0.0%) in Afar and Benishangul Gumuz regions (Table 2).

**Table 2:** Positivity rate of MTCT of HIV, regional frequency, and percentages

Variable	Frequency (%)	95.0% CL (Lower—Upper Count)	Negative (%)	Positive (%)
Positivity rate of MTCT of HIV			5533(97.4)	146(2.6)
Region of test				
Addis Ababa	3239 (57)	(3166–3312)	3166(97.7)	73(2.3)
Afar	09 (0.2)	(4–16)	9(100)	0(0)
Amhara	197 (3.5)	(171–225)	193(98)	4(2)
Benishangul Gumuz	06 (0.1)	(3–12)	6(100)	0(0)
Gambella	466 (8.2)	(427–508)	450(96.6)	16 (3.4)
Oromia	1055 (18.6)	(998–1113)	1014(96.1)	41 (3.9)
SNNPR	75 (1.3)	(60–93)	74(98.7)	1 (1.3)
Tigray	632 (11.1)	(587–680)	621(98.3)	11 (1.7)

*CL* Confidence Limit, *MTCT* Mother to Child Transmission, *SNNPR* Southern Nations, Nationalities, and Peoples’, Region, *HIV* Human Immunodeficiency Virus

### Trends of HIV infection among HEI over five years period

**Fig. 2:**
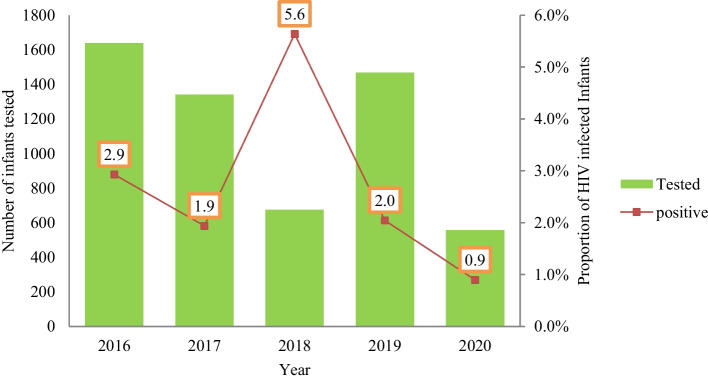
HIV positivity rate, and trend of MTCT in the past five years in Ethiopia, from 2016 to 2020

The positivity rate of HIV infection decreased from 2.9% in 2016 to 0.9% in 2020. However, the positivity rate of HIV infection among infants increased in 2018 (5.6%) (Fig. 2).

### Factors associated with HIV infection

Being tested after 6 weeks of age, absence of PMTCT service, nevirapine prophylaxis not obtained, and unknown ART status of the mother during delivery were significantly associated with HIV infection in bivariate analysis (Table 3). However, being tested after 6 weeks of age *p* < *0.001*, absence of PMTCT service *p* = *0.001*, nevirapine prophylaxis not obtained *p* < *0.001,* and born from mothers with unknown ART status *p* < *0.001*, and newly initiated ART during delivery *p* < *0.001* were continued to be significantly associated with HIV infection after adjusting for potential confounders (Table 3).

**Table 3:** Factors associated with HIV infection 2016–2020

Variables	EID PCR	Test results	COR (95% CI)	*P*–value	AOR (95% CI)	*P–*value
	Negative (%)	Positive (%)				
Sex						
Female	2683 (97.3)	75 (2.7)	1	0.492		
Male	2850 (97.6)	71 (2.4)	0.9(0.6–1.2)			
Age group						
Referred at ≤ 6 weeks	3166 (98.8)	40 (1.2)	1		1	
Referred > 6 weeks	2367 (95.7)	106 (4.3)	3.5(2.5–5.1)	< 0.001	2.7(1.8–4.0) ^a^	< 0.001
Infant feeding Practice						
Exclusive breast feeding	4041(97.4)	109 (2.6)	1			
Replacement feeding	1492 (97.6)	37 (2.4)	1.1(0.8–1.6)	0.663		
Intervention given to infant in PMTCT						
Present	5317(98.1)	103(1.9)	1		1	
Absent	216(83.4)	43(16.6)	10.3(7.0–15.0)	< 0.001	4.6(2.9–7.4) ^a^	0.001
Infant received nevirapine prophylaxis therapy						
Yes	5062 (98.0)	104 (2.0)	1		1	
No	464 (90.4)	49 (9.6)	5.5(3.9–7.9)	< 0.001	2.0(1.3–3.2) ^a^	< 0.001
Mother ART status						
Previously on ART	1445(97.8)	33(2.2)	1		1	
Newly initiated up on delivery	4057(97.9)	88(2.1)	3.6(2.5–6.2)	< 0.001	12.9(6.1–26.9) ^a^	< 0.001
Status not known	31(55.4)	25(44.6)	3.4(2.2–5.5)	< 0.001	11.0(5.5–22.1) ^a^	< 0.001

*EID* Early Infant Diagnosis, *PCR* Polymerase Chain Reaction, *COR* Crude Odds Ratio, *AOR* Adjusted Odds Ratio, *PMTCT* Prevention of Mother-to-Child-Transmission, *ART* Antiretroviral Therapy

^a^ Statistically significant

## Discussion

This study aimed to determine the positivity rate of HIV in HEI, five years trend of MTCT of HIV, and factors that are associated with HIV infection among HEIs whose specimens referred to EPHI for EID. The current study revealed that five-year positivity rate of HIV in HEIs was 2.6%. The positivity rate of MTCT was high in 2018; however, the overall trend was shown deceasing over the past five years. Being tested after 6 weeks of age; absence of PMTCT service; nevirapine prophylaxis not obtained, and unknown ART status of the mother during delivery were significantly associated with HIV infection among infants born from HIV-positive mothers.

The current study showed the positivity rate of HIV infection among infants born from HIV-positive mothers is higher than the WHO’s zero new HIV infection in infants born from HIV positive mothers by 2020 recommendation [5]. However, the positivity rate of HIV in infants born from HIV-positive mothers was nearly similar with the prevalence indicated in the Joint United Nations Programme on HIV/AIDS (UNAIDS) WHO strategy on HIV elimination which is 2.0% or less than among infants who are non–breastfeeding by 2020 [23]. In addition, the UNAIDS 2016 start free, stay free, and AIDS free MTCT of HIV is 2.8% at six weeks from data reported from 23 country which also include Ethiopia [23]. This result is similar with the present study estimation (2.9%) for 2016. Furthermore, the prevalence of start free, stay free, and AIDS free in 2018 was (2%) in Ethiopia [24] which is lower than the current study finding (5.6%). This could be due to the exclusion of many cases from analysis in 2018 due to data incompleteness. In contrast, the prevalence of HIV in infants at six weeks after birth was 9% in 2019 [25], while 8% in 2020 [26]. These results are higher compared to 2% in 2019 and 0.9% in 2020 in the current study. This difference could be due to few records were retrieved from the database.

In contrast to our finding, a previous study reported from Adama, central Ethiopia, indicated a lower (0.4%) prevalence of MTCT of HIV [27] than the current study. This difference is most probably due to the study population, sample size and study design. In the case of a study reported from Adama, the study design was a retrospective cohort that sampled based on the exposure status, while in the present study the study design was cross-sectional and the sample was all specimens referred to the national laboratory for EID test. Moreover, few children were included to the current study analysis from Adam, because majorly of the children in Adam access laboratory test from Adama regional laboratory and not referred to national laboratory unless service interrupted.

Other studies from different parts of Ethiopia have shown higher HIV prevalence among infants born from HIV-positive mothers [16, 28–35]. A study reported from Addis Ababa reported double prevalence (4.2%) of HIV infection in HEI [19] of the current study. This difference might be due to the sample size, study period, PMTCT service accessibility and strength. A study from Jimma, southwest Ethiopia has also indicated the highest prevalence of HIV infection among HEI 17% [36]. This difference might be because of the differences in study period, PMTCT strategies during the study period, sample size and data source. The data source in the study reported from Jimma was patients’ treatment care at health facilities, and the data collection period was older than the present study period. However, the data source for our study was the national EID database which contains the result of infants’ specimens referred to national laboratory for referral service.

The trend of HIV positivity rate in HEI has shown a decreasing tendency across the past five years. However, the highest positivity rate (5.6%) was seen in 2018. This could be due to the exclusion of a lot of cases from analysis due to data incompleteness in 2018. The declining trend of HIV infection across the five-year is similar to a study reported from China in which the trend showed a 48.5% reduction from 2014 through 2019 [37]. Moreover, a study reported from Vietnam indicated a declining trend of HIV MTCTfrom 27.9% in 2007 to 0% in 2018 [11]. Similarly, a study reported from Burkina–Faso indicated that decreasing trend of HIV infection proportion which decreased from 10.4% in 2006 to 0.0% in 2015 [12]. Moreover, a study reported from Kenya showed a declining trend of HIV MTCTfrom 19.8% in 2007 to 7.2% in 2013 [13]. These studies’ results are similar with the present study findings in which the trend of HIV positivity rate among HEIs was significantly shown decreasing tendency.

In the present study being tested for HIV after 6 weeks of age significantly increased the odds of HIV vertical transmission. This finding is similar with the findings reported previously from different countries [18, 30, 35, 38]. Similarly, a finding reported from Kenya indicated that infants tested after 6 weeks of age had a 6.3 times higher odds of getting HIV infection than those tested before 6 weeks [28]. The possible explanation for the high positive rate among infants tested after six weeks of age might be due to lack of prophylaxis, ARV interruption history of the mother which could increase viral load and leads to transmission, and increased exposure time after birth. In contrast, a study reported from Dire Dawa Eastern Ethiopia showed that age at a test of infants was not significantly associated with MTCT of HIV [14]. This difference might be due to the small sample size used by the study reported from Dire Dawa [14].

It was found that infants who did not receive PMTCT service had 4.6 times higher odds of HIV infection than those who received the service in the current study. This result is in-line with the finding reported by Wudineh et al., 2016 [14]. In addition, in the present study, infants who did not receive nevirapine prophylaxis had 2 times higher odds of HIV infection compared to those who obtained the prophylaxis. This result is similar with reports from different settings [11, 14, 18, 19, 32, 35]. A systematic review and meta-analysis pooled data from different setups also showed that infants on nevirapine prophylaxes were at lesser risk of HIV infection than those who missed the service [21, 31]. It suggests that nevirapine prophylaxis could prevent MTCT of HIV during breastfeeding through viral suppression.

Unknown ART status of the mother during delivery and newly ART initiation at delivery were significantly associated with HIV infection among HEI in the current study. This is consistent with previously reported studies from different areas [11, 14, 28, 32, 36–38]. This ascertains that mother’s early initiation of ART has an effect on viral suppression which in terns to the prevention of HIV transmission from mother to infant during pregnancy, delivery and breastfeeding.

### Limitations of the study

This study enrolled large sample size covered large geographic area of the country and allows stable estimates of the positivity rate of HIV in infants born from HIV-positive mothers and determines factors associated with transmission. Nevertheless, this study had some limitations. As the data for this study was collected from existing EID database, missing some variables such as duration on ART, CD4 count, number of mothers delivered and other clinical information is inevitable. There were also data points missing that could increase or decrease potential risk factors. Furthermore, the results of this study cannot be generalized to all regions of the country because referral of the children to national referral laboratory could not be random. These limitations might be underestimated the proportion of MTCT and the effect of factors associated with MTCT of HIV due to the included infants are not represent all HEIs in the country.

## Conclusions

The positivity rate of MTCT of HIV is declining steadily in Ethiopia. However, considerable proportions of infants are still acquiring HIV from their mothers either during pregnancy or delivery or breastfeeding. Thus, strengthening PMTCT service, early HIV screening and initiation of ART for pregnant mothers, and early infant diagnosis are important to achieve nationally and internationally set targets to reduce MTCT of HIV.

## Data Availability

The data used for this study can be available from the corresponding author on reasonable request and with the permission of EPHI.

## Abbreviations

AIDS: Acquired Immunodeficiency Syndrome
AOR: Adjusted Odds Ratio
ART: Antiretroviral Therapy
ARV: Antiretroviral
CI: Confidence Interval
COR: Crude Odds Ratio
DBS: Dried Blood Spot
DNA: Deoxyribonucleic Acid
EID: Early Infant Diagnosis
EPHI: Ethiopian Public Health Institute
HEI: HIV-Exposed Infant
HIV: Human Immunodeficiency Virus
MTCT: Mother -To-Child Transmission
PCR: Polymerase Chain Reaction
PMTCT: Prevention of Mother-To-Child Transmission
RNA: Ribonucleic Acid
UNAIDS: Joint United Nations Programme on Human Immunodeficiency Virus/Acquired Immunodeficiency Syndrome
WHO: World Health Organization
